# Evaluating Microsoft Copilot in Qualitative Health Research: Accurate for Manifest Content Coding but Limited in Latent Interpretation

**DOI:** 10.7759/cureus.95719

**Published:** 2025-10-29

**Authors:** Maria Lund-Tonnesen, Susanne Vahr Lauridsen, Jacob Rosenberg

**Affiliations:** 1 Department of Surgery, Herlev Hospital, Herlev, DNK

**Keywords:** analyzing data, artificial intelligence (ai), content analysis, microsoft copilot, qualitative research

## Abstract

Introduction

Artificial intelligence (AI) is becoming more integrated in different research assignments, and this ongoing development opens opportunities to optimize resources, e.g., using AI in resource-intensive and time-consuming tasks like qualitative analysis of interview data. We aimed to test if Microsoft’s Copilot could perform a content analysis on interview data using Graneheim and Lundman’s method comparable to human analysis.

Methodology

We used a company-protected version of Microsoft’s AI-powered assistant Copilot, which is based on large language models. The company-protected Copilot version ensured data security. A manual analysis of six interviews was conducted before this study using Graneheim and Lundman’s method of content analysis. We conducted four analyses using Copilot and compared the results with those obtained through manual analysis. Copilot was prompted to use Graneheim and Lundman’s method, and we tried providing it with an objective and a context.

Results

When prompted to use Graneheim and Lundman’s method, Copilot was able to perform content analyses with high resemblance to the manual one, especially in terms of selecting meaningful units, as well as when coding them, which is within the descriptive analysis. It could also create subthemes and overarching themes resembling the manual ones; however, the interpretive analysis lacked nuances compared to the manual one. Copilot produced more accurate manifest content when only given Graneheim and Lundman’s method. When given the objective, the analysis was shorter with fewer meaningful units. When given the context of the interviews, Copilot over-interpreted, and the analysis was mainly descriptive.

Conclusions

Copilot was able to perform a content analysis very similar to the manual one regarding the descriptive analyses on the manifest content using Graneheim and Lundman’s method. However, it’s interpretation of latent content lacked nuance - a limitation Copilot itself acknowledged. Copilot performed best when guided by the methodological framework alone, rather than the study’s objective or context. While content analysis remains, a co-creative process requiring manual input, especially during interpretation, Copilot shows promising potential in supporting the early stages of analysis focused on manifest content.

## Introduction

Artificial intelligence (AI), particularly models designed to process and generate human language, known as large language models (LLMs), is increasingly being applied in health research [1,2], for example, in research formality [3], diagnostics [4], statistics [5], and interview transcription [6]. We see great potential in using AI within work areas that are time-consuming and resource-intensive like qualitative interview analysis. Typically, interviews are guided by an interview guide, recorded, and then transcribed-either manually or with AI [7]. The interaction between the interviewer and participant during the interview will be unique and co-create the data [8]. Transcription is the first step in analysis, helping researchers understand the data [9]. Once all interviews are transcribed, researchers analyze them using methods like content analysis or systematic text condensation [9,10]. Ideally, two or more researchers work together to improve trustworthiness and reduce bias [11]. A clear methodological framework is important for transparency and credibility. Therefore, using a methodological framework substantiates trustworthiness in both the analytical process and the findings [11]. Since qualitative analysis is based on language, it makes sense to explore whether AI can assist. Microsoft’s Copilot was chosen because a company-protected version was available in the Hospital. Using the company-protected version helped secure data because it does not store data for training and learning, unlike public AI. Dealing with interview data, it is important to secure the anonymity of the people interviewed and to store data according to General Data Protection Regulation (GDPR) [12]. Our chosen methodological framework was Graneheim and Lundman’s content analysis, a qualitative approach that involves identifying meaning units, coding, and developing themes to analyze both manifest and latent content [9]. When doing a content analysis, you move between the manifest content, which is descriptive, and the latent content, which is interpretive [9]. The basis of content analysis is an understanding of data being co-created by the interviewer and participant, and interpretation in the analysis being co-created by the researcher and the text, making it dependent on context and subject [8]. Graneheim and Lundman’s method was chosen as it was the one used for the manual analysis.

Other studies have tested AI tools like ChatGPT [13-21], Gemini [14,16,22], and Claude [14,16] for qualitative analysis, but to our knowledge, not Microsoft’s Copilot. Using AI can raise some ethical concerns, as data security may be compromised; this is an essential aspect to consider. On this background, we aimed to test whether a company-protected version of Copilot could perform content analysis based on Graneheim and Lundman’s method on interview data corresponding to a human manual analysis.

## Materials and methods

Copilot is an AI-powered assistant developed by Microsoft. It is based on machine learning, like other chatbots, and develops and learns through interaction. Copilot offers contextual assistance, here amongst data analysis [23]. The version of Copilot used in this study was a company-protected solution for employees in the Capital Region in Denmark. In the company-protected solution, questions and results are not used for learning, and they are not stored opposite public generative AI. To test Copilot in the present study, we used transcripts from six interviews collected for an implementation study. Part of the aim of the implementation study was to explore the use of the Measure Yourself Medical Outcome Profile 2 (MYMOP2) [24] among healthcare professionals. We identified three themes across the six interviews: (1) Preparing for consultation, (2) Use of MYMOP2 creating value in consultation, and (3) Thoughts on implementation. The participants were six healthcare professionals who, before the interviews, gave written consent allowing AI analysis of the interview transcripts. The six healthcare professionals were the only staff connected with the Sequelae Center and therefore the accessible sample. Interviews were conducted in March 2025 with a median duration of 18 minutes (range 10-28). The interviews were transcribed using AI [6]. MLT listened through all interviews while correcting as advised when transcription is done by someone else, to ensure that the transcription is verbatim [25]. The interview data were analyzed by MLT and SVL before this study, following Graneheim and Lundman’s method for content analysis [9]. The authors analyzed independently, and disagreements were discussed until consensus. Both MLT and SVL are experienced in doing qualitative analysis. As the interviews were conducted in Danish, they were translated into English in Copilot with all personal information deleted beforehand. The initial manual analysis was used as a comparison to those done by Copilot, and when comparing the manual analysis, it served as the *gold standard*. We used three categories when comparing: green, indicating that the quote could be found in both manual and Copilot analysis; yellow, indicating that the quote covered the same meaning as other quotes in the manual analysis; and red, indicating that neither the quote nor its meaning could be found again in the manual analysis. The four Copilot analyses were systematically color coded one by one, examining each meaning unit, comparing it with the manual analysis. 

Graneheim and Lundman work with the assumption that there are two types of content in a text - manifest and latent. Manifest content is the literal and obvious content in the text, where latent content refers to the underlying meaning, which is derived from interpretation. They present different concepts, which can be used when performing a content analysis. Here, we present the ones used when analyzing manually [9]:

*The units of analysis:* six interviews about implementing MYMOP2

*Meaning units:* words, sentences, or paragraphs containing aspects related to each other through their content and context

*Condensation:* shortening the meaning units while preserving the essential meaning

*Abstraction: *identifying patterns by coding the meaning units and creating themes

In the manual analysis, we identified meaning units, condensed them, and created codes and subthemes within each interview. We then conducted a cross-case analysis of all interviews, resulting in three overarching themes. When reading the complete text, identifying meaning units, condensing them, and developing codes or categories, you work mainly within the manifest content, which is descriptive. When identifying subthemes and themes, you work more with the latent content, which is to be interpreted [8]. It is essential to go back and forth in the process, ensuring that the latent content aligns with the manifest content [9]. Both manifest and latent content require interpretation, but the depth and level of abstraction vary [8]. Before starting, we asked Copilot if it was familiar with Graneheim and Lundman’s method of content analysis. This was done to secure methodological consistency. Copilot was familiar with the process and referred to the exact steps of analysis. We repeated the Copilot analysis four times. The prompts are shown in Table 1.

**Table 1:** Prompts used for the four Copilot analyses. MYMOP2, Measure Yourself Medical Outcome Profile 2

Analysis	Procedure
Copilot analysis 1	Prompt: Can you do the following, using the uploaded transcript? You have previous experience doing qualitative research. Here is the transcript of an interview with a participant in a research project. Can you complete a content analysis?
Copilot analysis 2	Prompt: Can you do the following, using the uploaded transcript? You have previous experience doing qualitative research. You work with content analysis based on transcribed interviews, following Graneheim and Lundman’s method. Here is the transcript of an interview with a participant in a research project. Read the text carefully and complete the following tasks: The first step is reading the transcript to gain a sense of data as a whole. Afterwards you read and identify quotes categorizing them as meaningful units. In each interview, these will be condensed into shorter sentences and be coded.
Copilot analysis 3	Prompt: Can you do the following, using the uploaded transcript? You have previous experience doing qualitative research. You work with content analysis based on transcribed interviews, following Graneheim and Lundman’s method. Here is the transcript of an interview with a participant in a research project. Read the text carefully and complete the following tasks: The first step is reading the whole transcript to gain a sense of data as a whole. Afterwards you read and identify quotes categorizing them as meaningful units. In each interview these will be condensed into shorter sentences, assigned a code, and subthemes will be developed. The research objective is to support the implementation by exploring the experiences of using MYMOP2 among healthcare professionals in the Sequelae Center during the implementation process.
Copilot analysis 4	Prompt: Can you do the following, using the uploaded transcript? You have previous experience doing qualitative research. You work with content analysis based on transcribed interviews, following Graneheim and Lundman’s method. Here is the transcript of an interview with a participant in a research project. Read the text carefully and complete the following tasks: The first step is reading the transcript to gain a sense of data as a whole. Afterward, you read and identify quotes categorizing them as meaningful units. In each interview, these will be condensed into shorter sentences, assigned a code, and subthemes will be developed.

**Figure 1:**
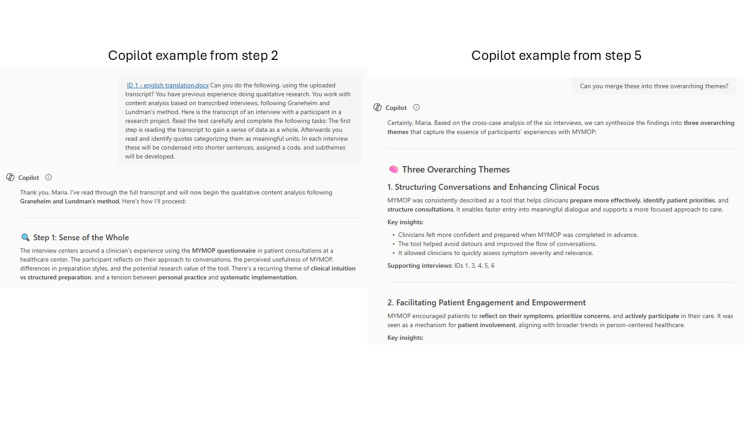
Copilot screenshots from the fourth Copilot analysis.

In the first Copilot analysis, we did not specify the methodological frame but asked it to do a content analysis. This was done to test the importance of having a common methodological framework when working with qualitative content analysis. In the second analysis, we were not specific enough in our prompt, and we received categories instead of subthemes. This was addressed in the third prompt, as shown in Table 1. In the second analysis, we attempted to provide Copilot with context after analyzing all six interviews. We prompted: How would the analysis look if I told you that these interviews are with healthcare professionals (doctors, nurses, and a dietician) working in a sequelae center treating patients with colorectal and anal cancer? In the third analysis, Copilot was given the research objective from the original study's interviews. In the fourth and final analysis, neither objective nor context was added. All results from Copilot were copied and stored on a secure data drive hosted by The Capital Region of Denmark. The steps of the fourth Copilot analysis, without context or objective, are shown in Table 2 and screenshots from steps two and five are shown in Figure 1.

**Table 2:** Steps of analysis in Copilot using Graneheim and Lundman’s method.

Steps of analysis in Copilot
Step 1. We uploaded the transcript as a document as it would otherwise exceed the allowed number of characters.
Step 2. We used the prompt: Can you do the following, using the uploaded transcript? You have previous experience doing qualitative research. You work with content analysis based on transcribed interviews, following Graneheim and Lundman’s method. Here is the transcript of an interview with a participant in a research project. Read the text carefully and complete the following tasks: The first step is reading the transcript to gain a sense of data as a whole. Afterwards you read and identify quotes categorizing them as meaningful units. In each interview these will be condensed into shorter sentences, assigned a code, and subthemes will be developed.
Step 3. Copilot provided an example of a coding table divided in four subthemes; we prompted: Have you analyzed the entire interview? Otherwise please do so. Copilot now provided a coding table with five subthemes. Steps 1-3 were repeated on the remaining five interviews.
Step 4. To find overarching themes we used the prompt: Can you conduct a cross-case analysis from the six interviews identifying themes to be presented? Copilot presented six themes with key insights under each theme and participant ID numbers to support these.
Step 5. As we manually identified three overarching themes, we prompted Copilot: Can you merge these into three overarching themes? Copilot then produced three themes with key insights under each theme also supported with participant ID numbers.
Step 6. Lastly, we prompted: Can you conduct a case cross analysis from the six interviews identifying both subthemes and themes to be presented? Copilot then presented three themes not identical in wording, but with the same elements substantiated by subthemes, description of subthemes and participant ID numbers.

## Results

**Figure 2:**
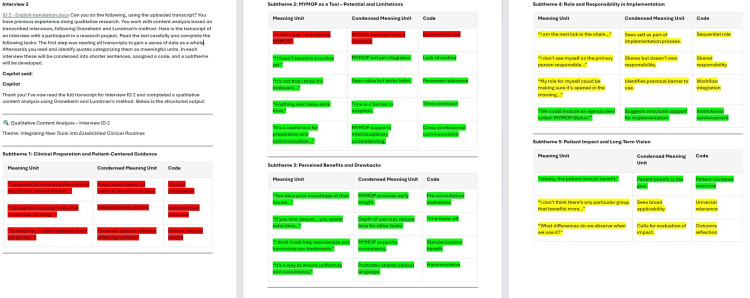
Color coding of interviews from the fourth Copilot analysis. Color coding of interviews after step 3 was completed: green highlighting indicates a quote found in both the manual and Copilot analyses; yellow highlighting indicates a quote covering the same meaning as other quotes in the manual analysis; red highlighting indicates a quote or meaning not found in the manual analysis.

There was a high degree of consensus between the analytic steps in the manual analysis and Copilot analyses 2-4. However, the fourth Copilot analysis most closely resembled the manual analysis, as it provided the most meaningful units, had subthemes instead of categories, and focused more on latent content. The color coding of an interview from the fourth Copilot analysis is illustrated in Figure 2. The red parts represented meaning units, which we found irrelevant for answering our research question.

**Figure 3:**
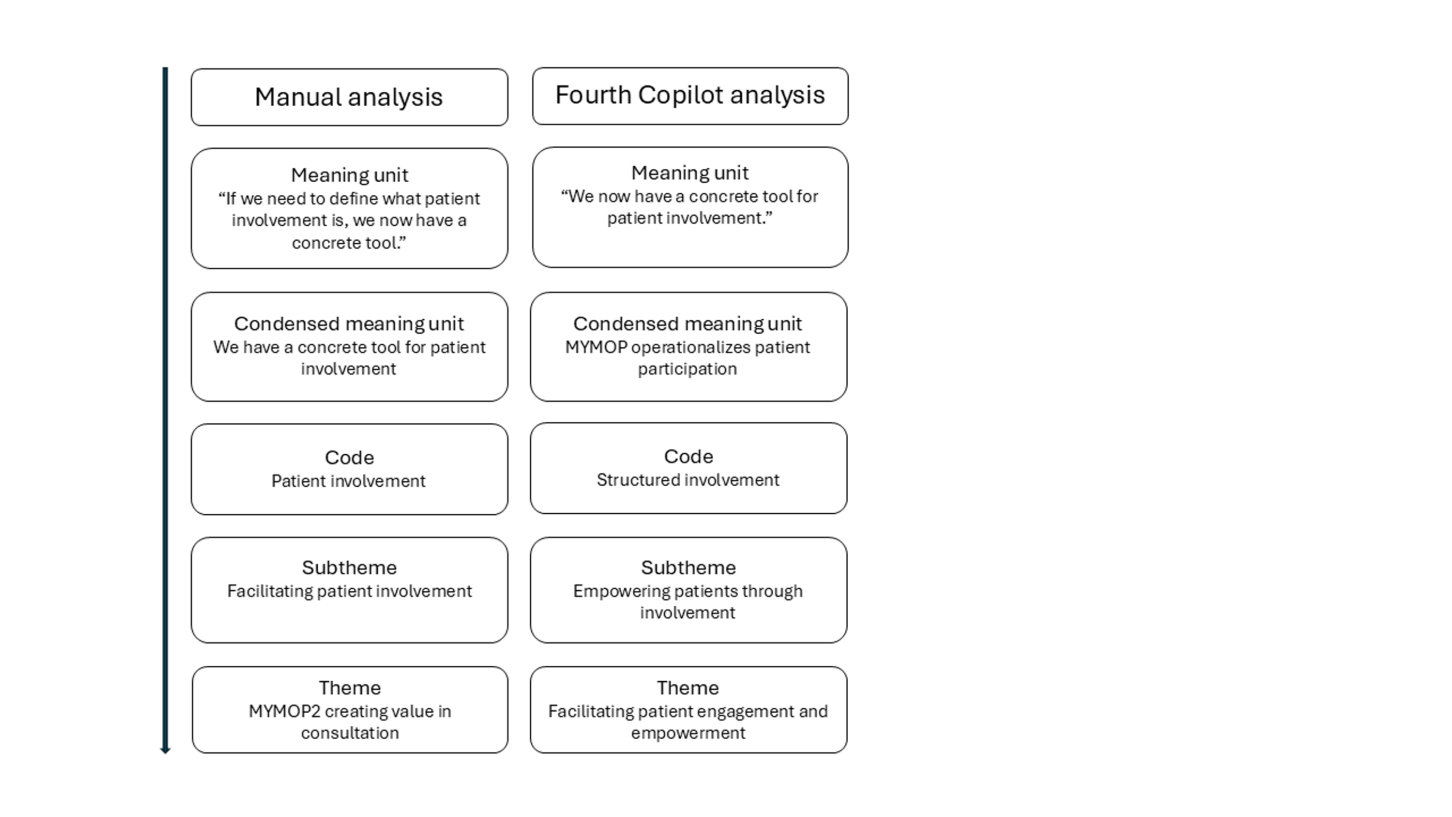
Coding tree example from the manual analysis and the fourth Copilot analysis.

Within the manifest content, the meaning units were quite similar, as illustrated in Figure 3. Copilot rephrased some of the meaning units, which is not in line with Graneheim and Lundman’s method [9]. The condensed meaning units and codes were also similar in terms of meaning, although not necessarily in wording. However, the manual analysis proved more detailed, with more meaningful units, than any of the analyses done by Copilot. An example of a coding tree is shown in Figure 3. 

When asked to perform a content analysis without a methodological framework, Copilot produced an analysis, the elements of which are presented in Table 3.

**Table 3:** Elements of the first Copilot analysis without a methodological framework.

Research context	Emerging themes	Interpretation and insights	Conclusion	Summary table
Copilot contributed suggestions related to the tool under discussion, the setting, and the participant profile.	Five to six themes were described for each interview.	Copilot produced interpretations with three to four insights per interview.	This was only provided for the first interview.	Copilot asked whether we wanted summary tables, which included representative quotes, descriptions of the quotes, and themes.

There was overlap with both the manual analysis and the other Copilot analyses; however, it was shorter and less detailed, primarily focusing on manual content rather than the latent content. Regarding the three overarching themes, these are shown in Table 4. It is worth mentioning that the themes from the manual analysis were not based solely on the six interviews. When the manual analysis was conducted, it also included six prior interviews with the healthcare professionals and 12 patient interviews, which also influenced the content and wording of the themes. Despite the wording being different, the essential content was the same. Theme 1 focused on how preparing with the structured questionnaire (MYMOP2) gave focus to the consultations. Theme 2 focused on how involving patients created value, as both healthcare professionals and patients were better prepared, and because patients set the agenda. Theme 3 focused on the implementation, specifically how the healthcare professionals perceived their own role, identified barriers, and sustained its use. The third copilot analysis was different from the others. Here, the implementation theme was separated into two, which is probably because this analysis included the objective concerning implementation.

**Table 4:** Overarching themes from all analyses.

Themes	Copilot 1	Copilot 2	Copilot 3	Copilot 4	Manual analysis
Theme 1	Structuring conversations and enhancing clinical preparation	Structuring conversations and enhancing clinical efficiency	Enhancing clinical communication	Structuring conversation and enhancing clinical focus	Preparing for consultation
Theme 2	Patient empowerment and engagement	Empowering patients through reflection and involvement	Implementation dynamics: integration, challenges, and sustainability	Facilitating patient engagement and empowerment	Use of MYMOP2 creating value in consultation
Theme 3	Implementation dynamics and sustainability	Navigating implementation challenges and sustaining use	Strategic value: research, quality improvement, and organizational fit	Implementation, integration, and sustainability	Thoughts on implementation

Providing Copilot with the context of the interviews was tested within the second Copilot analysis. Context was provided after the cross-case analysis, yielding three themes. The contextualized cross-case analysis somewhat resembled the preceding analysis, but with more interpretation, e.g., this statement: 

Copilot: *Given the intimate and often stigmatized nature of colorectal and anal cancer sequelae (e.g., incontinence, sexual dysfunction), the questionnaire encourages patients to reflect privately before the consultation, which may help them bring up difficult topics.*

This statement, saying that sequelae after colorectal and anal cancer often have an intimate and stigmatized nature, was not mentioned by any of the healthcare professionals and is therefore seen as an interpretation based on other available information from Copilot.

## Discussion

Copilot was able to complete a content analysis of six full interviews, using Graneheim and Lundman’s method as prompted. Compared with the manual analysis, there were many similarities, and the essential meaning found in the manual analysis was preserved. However, all the Copilot analyses were shorter, less detailed, and focused mainly on the manifest content. In contrast, the manual analysis was more thorough, encompassing nuances and latent content not identified by Copilot.

Copilot demonstrated an ability to identify meaningful units; however, these were not consistently reproduced verbatim as prescribed by the method [9]. The codes, subthemes, and overarching themes identified by Copilot across the interviews were also somewhat similar to the themes found manually, not in wording, but in content and meaning. We view this as comparable to two researchers conducting collaborative data analysis, where the wording of codes, subthemes, and themes will also show variations that need to be negotiated. The most important aspect of collaborative analysis is finding meaning and reaching agreement about this [11]. There were different problems with the Copilot analyses. A significant issue was that Copilot rephrased data, thereby not accurately representing the participant’s statement, which could potentially lead to inaccurate results, as illustrated in Figure 3. In the first analysis, when not provided with a methodological framework, the content analysis became very superficial, as expected, underlining the importance of following a method. There were also problems concerning the level of interpretation seen when Copilot was given the interview context in the second analysis. Here, it made an interpretation that was not recognizable in the original data, either in wording or theme. It is problematic if the interpreted latent content cannot be traced back to the units of analysis, as this hampers methodological transparency [9]. When given the objective in the third Copilot analysis, the analysis presented was shorter, with fewer meaningful units, and overarching themes were not as aligned as in the other Copilot analyses.

Copilot was most accurate when it did not have context or a study objective. This is also not in line with Graneheim and Lundman, who argue that the analytic co-creation is also dependent on knowing the context of the interviews [8]. These limitations should be considered if using Copilot or other AI in qualitative analysis. Other studies analyzing qualitative data using AI technology have also concluded that AI can be effectively utilized to gain perspective, depth, and reduce bias; however, they also highlight limitations that necessitate human insight [13-15,22]. This is shown in our analysis with the distinction between analyzing the manifest and the latent contents. Also, the ethical considerations [16], like our considerations regarding data security, is mentioned as something to be aware of when employing this approach.

When asked about its own strengths and weaknesses, Copilot emphasized many of the same points that we have highlighted. AI is efficient and can cope with large datasets much faster than a human in terms of finding patterns, coding, and being consistent, which are valuable elements in the initial phase of analyzing manifest content. It can be easily documented, improving transparency, and it applies coding rules uniformly, which helps reduce human bias, such as confirmation bias. Limitations include losing nuances, as AI may lack the reflexive capacity of human researchers to critically engage with their own assumptions and positionality, thereby struggling to interpret latent content, emotional undertones, and cultural context - key aspects of Graneheim and Lundman’s approach. Copilot also emphasized the importance of prompting, with the possibility to enhance or reduce the quality of the outputs. Finally, Copilot also raised the ethical issues concerning data confidentiality in public AI. Copilot’s own advice for best practice when using with Graneheim and Lundman’s method was as follows:

(1) Utilize AI for preliminary coding or pattern detection but validate and refine the results manually.

(2) Combine inductive and deductive approaches - AI can assist with deductive coding if provided with a clear theoretical framework.

(3) Maintain human oversight throughout the process to ensure interpretive depth and ethical integrity.

This study has both strengths and limitations. A strength was that we utilized a company-protected Copilot solution, which secured our data and ensured ethical responsibility. Another strength was the use of a specific analysis method known to Copilot, which ensured the same methodological framework was applied to both the manual and Copilot analyses. We developed our prompt along the way, testing different variations that included the importance of methodological framework, objective, and context. Finally, we have specified our steps and prompts, making the process transparent. The study also has some limitations. As we used a company-protected version of Copilot to protect our data, we do not know how a public version would perform, as these are trained continuously, and there are even specific models available trained in qualitative research data analysis (e.g., available as Qualitative Research Data Analysis 4o in ChatGPT); one could assume that a public version continuously trained might perform better when analyzing latent content, especially AI made specifically for analyzing qualitative data. Another limitation is that we only tested Graneheim and Lundman’s method for content analysis, as this method was used in the manual analysis. It is unclear how Copilot would perform with another analysis method. When analyzing manually, there is a risk of coders' bias; however, employing multiple coders and a methodological framework for rigor help reduce this risk. The small sample size might also affect the transferability of the results to other settings. Lastly, the interviews were translated into English using Copilot. When translating, there is always a risk of losing meaning, as languages differ [25]. MLT reviewed all the translated interviews against the Danish transcripts, sentence for sentence, to ensure that the meaning was preserved. However, even though MLT is fluent in English, she is not a native English speaker, which might have affected the translation and, consequently, the results.

Based on this study and other similar studies, we see potential in using AI as a second part analyzer in the preliminary stages of qualitative content analysis, primarily because it will limit resources and enhance consistency and transparency in the analysis process. The next step could be using AI to apply the theory to the analysis.

## Conclusions

Copilot was used to perform a content analysis following Graneheim and Lundman’s method, similar to the manual approach conducted by two researchers. The strongest similarities between Copilot and the manual analysis were found in the interpretation of manifest content, where the meaning is explicit and directly observable. In contrast, the analysis of latent content, which requires deeper interpretive insight, showed greater variation, emphasizing that co-creation and human judgment are essential for this level of analysis. Based on these findings, we see potential in integrating Copilot into the early stages of qualitative research, specifically for identifying and organizing manifest content. We do, however, have some reservations as limitations were observed, such as Copilot generating incorrect interpretations when provided with interview context. Therefore, we recommend using Copilot and similar AI tools as supportive analytical partners in the initial phase of content analysis, while maintaining manual analysis to preserve the human perspective, particularly in interpretive tasks that AI is not yet equipped to handle, providing that local GDPR rules are followed.

## Appendices

**Table 5:** Interview guide

Interview guide	
Introduktion and purpose	
Repeat purpose of the interview and project in general Setting the frame for the interview	Thank you so much for being willing to share your experience with using MYMOP2 in connection with its implementation at Copenhagen Sequelae Center. The interview will take a maximum of 45 minutes, and I would like to inform you that it will be recorded for transcription purpose. Both the recording and the transcription will be treated confidentially and stored securely for 5 years. All participants will be anonymized. If you have any questions or uncertainties during the interview, please feel free to ask.
Research questions	
Background	Can you start by telling your name and your primary assignments at the Sequelae Center?
Usual practice	Try to describe one of the most recent conversations you've had at the Sequelae Centre. Can you try to describe your approach or give an example? How did you prepare yourself for the conversation? What did you do if the patient hadn’t filled out the MYMOP2 questionnaire in advance? How are you experiencing conversations with patients today compared to three months ago (before Christmas)? How do you feel the patients have responded to MYMOP2?
Use of MYMOP	Have you used MYMOP2 at the Sequelae Center? If not: why not? If yes: What is your impression of the questionnaire – has it changed since last time? Can you give an example? What do you see as advantages/disadvantages of MYMOP2 compared to your usual practice? What do you gain from using the MYMOP2 questionnaire today? What do you think patients gain from using the MYMOP2 form today?
Implementation	How do you see your role in the implementation of MYMOP2? Has that changed since last time? Who do you think benefits the most from MYMOP2? What impact do you think MYMOP2 will have on the Sequelae Center as a whole? What do you think will influence whether MYMOP2 is still being used in two years?
Summarize and finish	I am just going to summarize what we talked about You said that… is that correct? We are about to finish the interview do you have anything else you want to ad? Can I interview you again in two months if relevant? Thank you so much for your help, you are very welcome to contact me if you have further questions
